# Clinical characteristics and early prognosis of patients with SARS-CoV-2 infection undergoing joint arthroplasty during the COVID-19 pandemic

**DOI:** 10.1097/MD.0000000000026760

**Published:** 2021-08-20

**Authors:** Xin Jin, Mengcun Chen, Jinlong Wang, Shuhua Yang, Weihua Xu, Xianzhe Liu

**Affiliations:** Department of Orthopedics, Union Hospital, Tongji Medical College, Huazhong University of Science and Technology, Wuhan, People's Republic of China.

**Keywords:** COVID-19, hip replacement, joint arthroplasty, perioperative complications, SARS-CoV-2

## Abstract

The present study reported early clinical outcomes and perioperative precautions for medical staffs during joint arthroplasty procedures in SARS-CoV-2-infected patients.

The medical records of 8 patients with SARS-CoV-2 infection who underwent joint arthroplasty from January 19 to September 24, 2020 were retrospectively reviewed and analyzed. Perioperative precautions and follow-up (time length varies from 6 month to 13 months, 11 months in average) for SARS-CoV-2 infection of medical staffs were reported.

All patients recovered well from both the primary disease and SARS-CoV-2 infection. Significant improved Visual analogue scale was observed with no major complications or recurrence of the COVID-19 at discharge. There was no evidence indicating SARS-CoV-2 infection in any health providers.

Elective joint arthroplasties for patients in recovery period of SARS-CoV-2 infection could be continued under comprehensive preoperative evaluation and appropriate medical protection. For patients with currently confirmed or highly suspected COVID-19, the operation should be carried out only if it was essential.

## 1 Introduction

The present COVID-19 pandemic spread rapidly throughout the world. The pandemic has caused >1.3 billion infections and 2.9 million related deaths as the present study is prepared. Among these infections, some patients were health care providers and brought tremendous strike to the global medical system.^[1]^ Several institutions have issued recommendations that surgical procedures be restricted to emergency cases.^[2]^ However, during the post-outbreak period, all kinds of operations, including orthopedic operations, were increasing dramatically.^[3]^ Previous literature had reported guidelines for orthopedic procedures^[4]^ and strategies of proper personal protection equipment/gears for surgeons and anesthesiologists during the COVID-19 pandemic and provided useful information for the performance of the procedures.^[5–7]^

Joint arthroplasty is one of the most successful orthopedic procedures.^[8]^ For elderly patients with hip fractures, early joint arthroplasty can reduce mortality rate and complications associated with prolonged bed-ridden.^[9]^ However, during the COVID-19 pandemic, joint arthroplasty was classified as Tier-2 and was recommended to be canceled or postponed.^[2]^ It was reported that approximately 30,000 primary and 3000 revision hip and knee arthroplasty procedures would be canceled each week after excluding essential procedures during the COVID-19 restrictions.^[10]^ Joint arthroplasty has strict requirements for sterility since the peri-prosthetic joint infection (PJI) could result in disastrous consequences.^[11]^ The intraoperative blood loss and prolonged surgical time could also be a substantial restriction for the selection of patients.^[12]^ Besides, potential generation of aerosol caused by osteotomy, bone reaming, and prosthesis implantation required higher level of protection for the medical staff.^[13]^ Till now, there was still a paucity of data of perioperative administration and medical protections for joint arthroplasties in COVID-19-infected patients. Therefore, the present study retrospectively reviewed a series of patients with COVID-19 infection or in the recovery stage of previous infection who underwent joint arthroplasty during the COVID-19 pandemic. Early clinical outcomes and experiences of medical protection for the surgical and anesthesiology team were reported.

## 2 Materials and methods

### 2.1 Study design and participants

A retrospective review of the medical records of patients with COVID-19 infection in Wuhan Union Hospital and Hubei Hospital of Traditional Chinese Medicine was performed. From January 19 to September 24, 2020, 8 patients who underwent hip arthroplasty were involved. Written informed consent was obtained from all participants before enrollment in the present study. This study was approved by The Ethics Committee of Tongji Medical College, Huazhong University of Science and Technology (2020-S083). We analyzed all the data anonymously. The dataset for this study is available in Table 1. One patient was tested positive for COVID-19 and recognizable evidence of viral pneumonia on pulmonary computed tomography (CT) scan. One patient was tested negative for COVID-19 nuclear acid but presented typical clinical symptoms of COVID-19 and signs of viral pneumonia on CT scan. The blood serum SARS-CoV-2 antibody test was IgM+/IgG+ as well. The patient was defined as a clinically diagnosed COVID-19 case according to the New Coronavirus Pneumonia Diagnosis and Treatment Program (Trial Seventh Edition), published by the National Health Commission of China.^[14]^ The other 6 patients were tested negative for COVID-19 and IgM-/IgG+. None of them had clinical or radiographic indications of COVID-19 and thus were recognized as previously COVID-19 infected cases in the recovery stage.^[15]^

**Table 1:** Clinical characteristics of patients underwent joint arthroplasty^∗^.

	Case 1	Case 2	Case 3	Case 4
Clinical characteristics				
Sex, age, y	F, 79	M, 74	F, 27	F, 77
Orthopaedic diagnosis	Femoral neck fracture	Intertrochanteric fracture	Osteoarthritis	Femoral neck fracture
Epidemiological history	Yes (exposure to relevant environment)	Yes (exposure to relevant environment)	Yes (exposure to relevant environment)	Yes (exposure to relevant environment)
Complications	Osteoporosis	Osteoporosis	None	Hypertension, diabetes, osteoporosis
Signs and symptoms				
Fever	Yes	No	No	No
Cough	Yes	No	No	No
Fatigue	No	Yes	No	No
Sore throat	No	No	No	No
Dyspnea	No	No	No	No
Chest pain	No	No	No	No
Nasal congestion	No	No	No	No
Headache	No	No	No	No
Dizziness	No	No	No	No
Diarrhea	No	No	No	No
Abdominal pain	No	No	No	No
Vomiting	No	No	No	No
Limited activity	Yes	Yes	Yes	Yes
Laboratory test results^†^				
White blood cell count (×10^9^ cells/L)	12.37 (↑↑)	12.91 (↑↑)	4.8	3.65
Neutrophil count (×10^9^ cells/L)	11.45 (↑↑)	10.82 (↑↑)	2.54	1.6 (↓)
Lymphocyte count (×10^9^ cells/L)	0.59 (↓↓)	0.9 (↓↓)	1.83	1.6
Monocyte count (×10^9^ cells/L)	0.33	1.19 (↑)	0.33	0.31
Platelet count (×10^9^ cells/L)	153	117 (↓)	205	180
APTT, s	31.2	35.4	38.9	39.2
PT, s	10.3 (↓)	14	13.8	13.6
d-dimer, mg/L	12.45 (↑)	2.36 (↑)	0.2	1.22 (↑)
Procalcitonin, ng/mL	0.219 (↑)	<0.13	<0.13	NA
CRP, mg/L	96 (↑↑)	29.81 (↑)	3.11	15.8 (↑)
ESR, mm/h	5	36 (↑)	2	2
Evidence of COVID-19				
SARS-CoV-2 quantitative RT-PCR	Positive	Negative	Negative	Negative
Serum IgM	NA	Positive	Negative	Negative
Serum IgG	NA	Positive	Positive	Positive
Typical signs of viral infection on CT	Bilateral	Bilateral	No	No
Treatments				
Operative procedure	Bipolar cementless hip hemiarthroplasty	Cementless total hip arthroplasty+plate	Cementless total hip arthroplasty	Bipolar cementless hip hemiarthroplasty
Oxygen inhalation	Yes	Yes	Yes	Yes
Antiviral therapy	Yes	Yes	No	No
Antibacterial therapy	Yes	Yes	Prophylactic	Prophylactic
Intravenous immunoglobulin therapy	No	No	No	No
Glucocorticoid therapy	Yes	No	No	No
LMWH (pre- /post-operation)	Yes/yes	Yes/yes	No/yes	Yes/yes
VAS (pre- /post-operation)	7/1	6/1	7/0	6/0
Caprini venous thromboembolism risk (pre- /post-operation)	Super high/super high	Super high/super high	Moderate/super high	Super high/super high
Complications				
Surgical wound infection	No	No	No	No
DVT	No	No	No	No
Dislocation	No	No	No	No

Case 5	Case 6	Case 7	Case 8
M, 85	F, 60	M, 70	F, 71
Intertrochanteric fracture	Femoral neck fracture	Osteonecrosis of the femoral head	Femoral neck fracture
Yes (exposure to relevant environment)	Yes (exposure to relevant environment)	Yes (exposure to relevant environment)	Yes (exposure to relevant environment)
Osteoporosis	Hypertension, osteoporosis	Hyperuricemia	Hypertension, osteoporosis
No	No	No	No
No	No	No	No
No	No	No	No
No	No	No	No
No	No	No	No
No	No	No	No
No	No	No	No
No	No	No	No
No	No	No	Yes
No	No	No	No
No	No	No	No
No	No	No	No
Yes	Yes	Yes	Yes
6.57	6.84	5.96	7.44
4.08	4.31	4.03	6.02
1.4	1.81	1.39	0.86
0.39	0.58	0.44	0.41
216	258	111 (↓)	189
43	28.6	31.4	33.1
15.8	12	12.8	13.7
0.79 (↑)	0.78 (↑)	NA	5.71 (↑)
3.57 (↑)	NA	NA	<0.13
3.14	7.33(↑)	3.11	49.6 (↑)
15	24 (↑)	13	16
Negative	Negative	Negative	Negative
Negative	Negative	Negative	Negative
Positive	Positive	Positive	Positive
No	No	No	No
Bipolar cementless hip hemiarthroplasty	Cementless total hip arthroplasty	Cementless total hip arthroplasty	Cementless total hip arthroplasty
Yes	Yes	Yes	Yes
No	No	No	No
Prophylactic	Prophylactic	Prophylactic	Prophylactic
No	No	No	No
No	No	No	No
Yes/yes	Yes/yes	No/yes	Yes/yes
7/1	6/0	5/0	8/2
Super high/super high	Super high/super high	Moderate/super high	Super high/super high
No	No	No	No
No	No	No	No
No	No	No	No

∗ DVT = deep vein thrombosis, LMWH = low-molecular-weight heparin, NA = not available, VAS = Visual Analogue Scale.

† White blood-cell count: normal range, 3.5 to 9.5 × 10^9^ cells/L, (↑↑) indicates leukocytosis (>10.0 × 10^9^ cells/L). Neutrophil count: normal range, 1.8 to 6.3 × 10^9^ cells/L, (↑↑) indicates neutrophilic granulocytosis (>7.5 × 10^9^ cells/L). Lymphocyte count: normal range, 1.1 to 3.2 × 10^9^ cells/L, (↓↓) indicates lymphopenia (<1.0 × 10^9^ cells/L). For the remaining laboratory values, the normal ranges are as follows: d-dimer, <0.5 mg/L FEU (fibrinogen equivalent units); procalcitonin, <0.05 ng/mL; C-reactive protein, 0 to 5 mg/L.; ESR, <20 mm/h for female, <15 mm/h for male. For those values, (↑) or (↓) indicates an increase or decrease compared with the normal level. CRP = C-reactive Protein, ESR = Erythrocyte Sedimentation Rate, APTT = Activated Partial Thromboplastin Time, PT = Prothrombin Time.

### 2.2 Workflow for patients with SARS-CoV-2 infection who need joint arthroplasty

**Figure 1:**
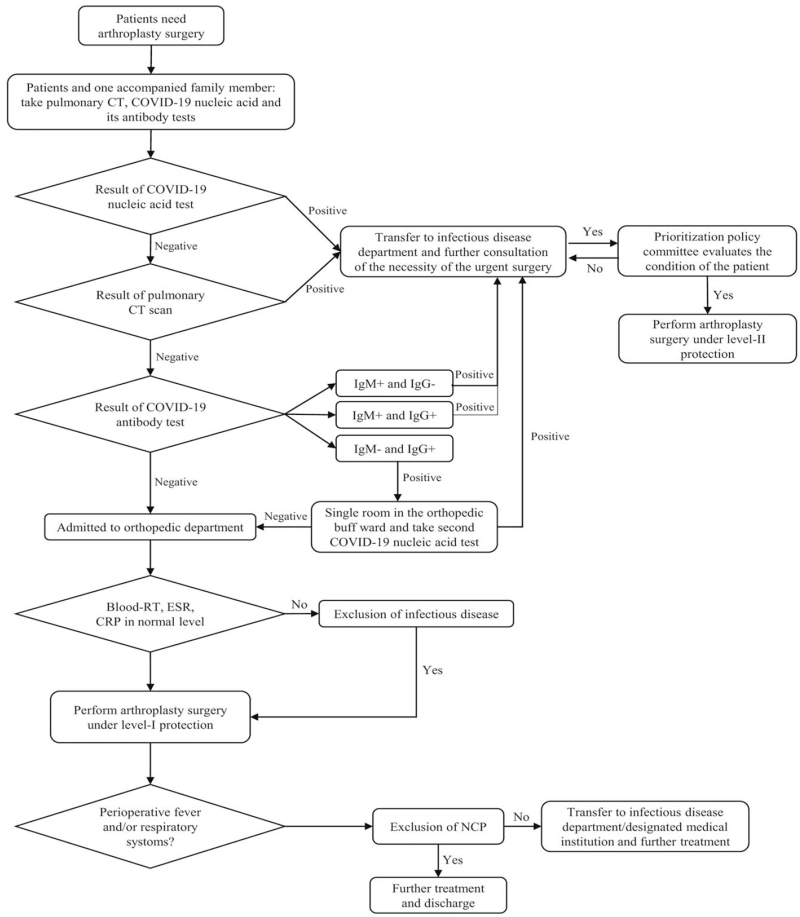
Flowchart for handling of a joint replacement surgery by orthopedic joint surgeon during COVID-19 epidemic period.

A prioritization policy committee was established to develop strategies for joint arthroplasty procedures during the COVID-19 pandemic.^[16]^ The detailed workflow was illustrated in Figure 1. The necessity and feasibility of the procedure were comprehensively appraised by the committee and conservative treatment was applicable for patients who were unsuitable (due to severe comorbidities or compromised pulmonary condition) or unwilling to receive the procedure. However, Table 2 demonstrated that classification of protection levels and suggestion of personal protective equipment for joint arthroplasty procedures in detail.

**Table 2:** Classification of protection levels for arthroplasty surgery.

Level of protection	Scope of application	Personal protective equipments
I	Suitable for pre-examination triage, outpatient clinics and performing arthroplasty surgery to patients in recovery stage of Covid-19.	Wearing a disposable working cap, surgical mask, face shield/googles, scrubs or working cloths, sterile surgical gowns and gloves if necessary, and washing hands thoroughly.
II	Medical staff like surgeon, engaging in diagnosis and treatment activities in close contact with suspected or confirmed patients.	Wearing a disposable working cap, medical protective mask, face shield/googles, medical protective clothing, sterile surgical gowns, sterile surgical gloves, shoe covers and double-gloving with the second pair of gloves covering the protective clothing sleeve, and washing hands thoroughly.
III	Medical personnel like anesthetist, who may be exposed to aerosol from suspected or confirmed patients due to tracheal intubation, tracheotomy, and so on.	When such personnel are working under the possibility of being sprayed or splashed with respiratory secretions or other substances, they should wear a disposable working cap, comprehensive respiratory protective device or positive pressure type head cover, medical protective mask, medical protective clothing, latex gloves, shoe covers and double-gloving with the second pair of gloves covering the protective clothing sleeve, and should wash their hands thoroughly.

**Figure 2:**
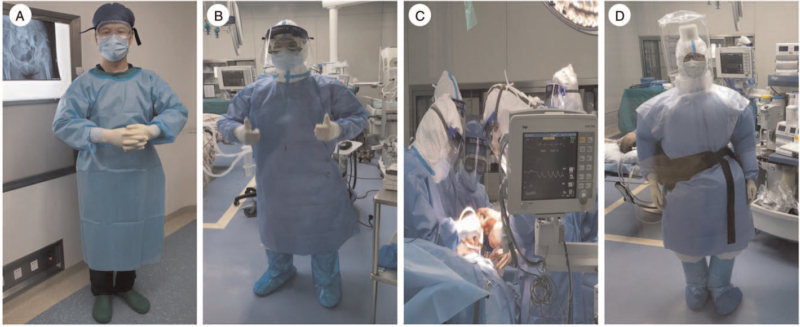
Personal protective equipment (PPE) used for arthroplasty surgery. (A) PPE used for protection level I. (B and C) PPE used for protection level II. (D) PPE used for protection level III.

Specifically, patients who were tested positive for COVID-19 nuclear acid, anti-SARS-CoV-2 IgM, or had typical clinical symptoms and viral pulmonary CT findings for COVID-19, would be transferred to an isolated room in the department of infectious disease and receive corresponding treatment before surgery. Only when the surgical intervention was recognized as emergent and the pulmonary condition was affordable for anesthesia should the patient be permitted to undergo the procedure. For these cases, the operating rooms (ORs) should have either a negative-pressure environment or an independent airflow system. The medical staff should wear Level-II personal protective equipment (PPE) (Fig. 2B/C). The anesthesiologist was advised to utilize Level-III PPE (Fig. 2D) with a positive-pressure headset to prevent spraying aerosols or droplets from the opened airway. However, if patients had only a positive SARS-CoV-2 IgG test with no typical clinical and radiographic findings, they were recognized as in the recovery stage of SARS-Cov-2 infection. After a second negative COVID-19 nucleic acid test over 24 hours, the patient could be admitted to an isolated room in the orthopedic ward. An elective joint arthroplasty could be continued for these patients, and the medical staff including anesthesiologist would utilize Level-I PPE including goggles or face shields (Fig. 2A).

### 2.3 Postoperative care and follow-up

After the procedure, confirmed or clinically diagnosed COVID-19-infected patients were admitted to the department of infectious disease for medical isolation. Patients in their recovery stage of COVID-19 infection were admitted to the orthopedic department for medical observation. Postoperative fever with/without respiratory symptoms should be considered as main criteria for isolation of patients immediately and comprehensively investigated with a pulmonary CT scan, a COVID-19 nucleic acid test, and a SARS-CoV-2 antibody test should be carried out instantly.

Laboratory tests and evidences of COVID-19 infection were monitored during the hospitalization. Visual analogue scales (VAS) and Caprini venous thromboembolism risk were used to assess perioperative pain and risk of thromboembolism. Low-molecular-weight heparin was used for DVT prophylaxis in all patients. Patients could be encouraged to mobilize and bear weight as tolerated with the assist of walker one day after the procedure. Abduction of the hip joint was recommended during the nursing process. Oral direct factor Xa inhibitor (apixaban) was used for the prevention of DVT after discharge.

Complications including wound conditions, infection, dislocation, and DVT were monitored during the hospital stay and at each follow-up (time length varies from 6 month to 13 months, 11 months in average). The medical records were provided by the 2 participating hospitals and were analyzed at the Department of Orthopedics, Union Hospital, Tongji Medical College, Huazhong University of Science and Technology, with use of a customized data-collection form.

### 2.4 Statistical analysis

SPSS software (version 23.0; IBM) was used for statistical analysis of primary data. Categorical variables are presented as the median and interquartile range or as the number and percentage. Diagrams of curves were drawn with use of Prism (version 8.3; GraphPad).

## 3 Results

**Figure 3:**
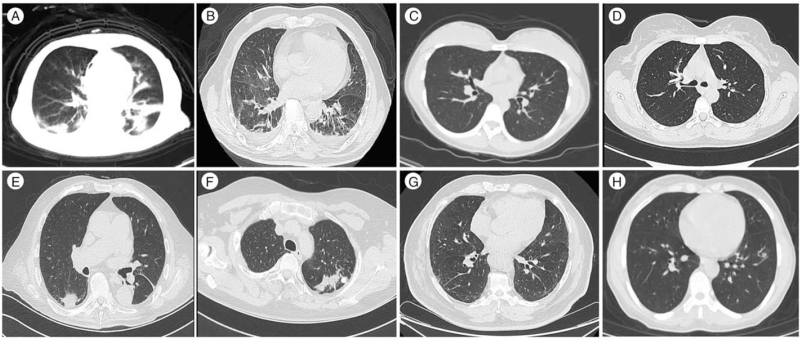
Chest CT scans for all 8 patients. (A and B) Case 1 and 2 had bilateral patchy consolidation and multiple ground-glass opacities. (C) Case 3, (D) Case 4, (E) Case 5, (F) Case 6, (G) Case 7, (H) Case 8.

The clinical characteristic of the eight patients (five women and three men) enrolled in the present study were presented in Table 1. Four patients were diagnosed as femoral neck fracture: 2 as intertrochanteric fractures, 1 as osteoarthritis of the hip and, 1 as osteonecrosis of femoral head. Given the results of the COVID-19 nucleic acid test, serum SARS-CoV-2 antibody test and pulmonary CT scan (Table 1 and Fig. 3), and according to the New Coronavirus Pneumonia Diagnosis and Treatment Program (Trial Seventh Edition). Case 1 was defined as the confirmed COVID-19 case, case 2 was the clinically diagnosed COVID-19 case, and the remaining six patients were deemed as in the recovery stage from COVID-19 infection.

**Figure 4:**
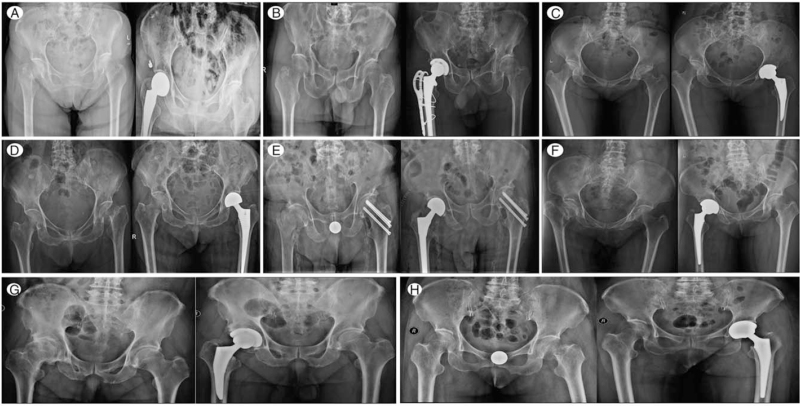
Anteroposterior (AP) pelvic radiographs of all 8 patients in the present study. (A) Case 1. AP pelvic radiographs showing a femoral neck fracture and postoperative radiograph. (B) Case 2. Preoperative and postoperative AP radiographs showing an intertrochanteric fracture. (C and D) Case 3 and 4. Preoperative and postoperative AP radiographs showing a femoral neck fracture. (E) Case 5. Preoperative pelvic radiographs showing an intertrochanteric fracture and postoperative radiograph. (F) Case 6. Preoperative and postoperative radiographs showing a femoral neck fracture. (G) Case 7. Preoperative and postoperative radiographs showing an osteonecrosis of the femoral head. (H) Case 8. Preoperative and postoperative radiographs showing a femoral neck fracture.

**Figure 5:**
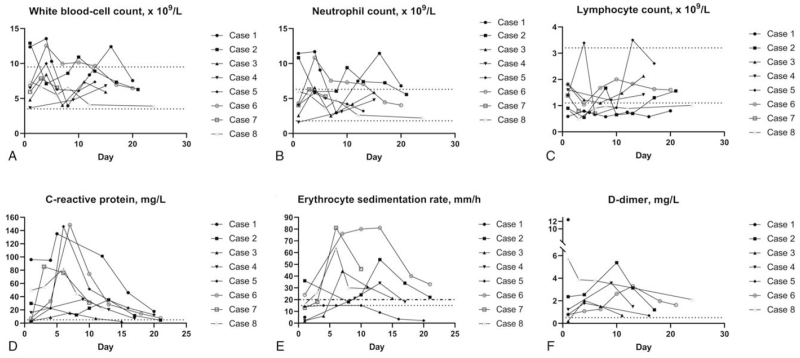
Line graphs illustrating detailed changes in white blood cell, neutrophil and lymphocyte count, CRP, ESR, and d-dimer for all 8 patients, starting on the day of admission. The normal ranges of laboratory test results are as follows: white blood-cell count, 3.5 to 9.5 × 10^9^ cells/L; neutrophil count, 1.8 to 6.3 × 10^9^ cells/L; lymphocyte count, 1.1 to 3.2 × 10^9^ cells/L; C-reactive protein, 0 to 5 mg/L; ESR <20 mm/h for female, <15 mm/h for male; d-dimer, <0.5 mg/L FEU (fibrinogen equivalent units).

Pre- and postoperative radiographic data of the procedure were illustrated in Figure 4. The results of laboratory tests are shown in Table 1 and Figure 5. Coagulation function was normal in all the 8 patients preoperatively. In confirmed and clinical diagnosed cases (Case 1 and 2), the level of white blood cell count, neutrophil count, C-reactive Protein (CRP), and d-dimer are higher than the normal threshold pre-operatively, whereas the lymphocyte count was lower than normal. In terms of Erythrocyte Sedimentation Rate (ESR), case 1 was in the normal range, whereas case 2 was higher than the normal threshold preoperatively. In the remaining 6 patients, the level of CRP and d-dimer were higher than normal in all fracture patients, ESR was higher in 2 patients. Two of the 6 patients had an elevated procalcitonin concentration. The detailed changes in white blood cell, neutrophil and lymphocyte count, CRP, ESR, and d-dimer were collected starting from the date of admission (Fig. 5). In general, those monitored markers increased rapidly after surgery in a short time, and then decrease slowly to normal range afterward.

All patients were managed with oxygen therapy. The confirmed or clinical diagnosed COVID-19 patients (Case 1 and 2) received antiviral (oseltamivir) and antibacterial therapy (moxifloxacin). The remaining 6 patients were treated with routine prophylactic antibiotic therapy (cefathiamidine). None of the patients were subjected to noninvasive ventilation, or intravenous immunoglobulin. Only case 1 received glucocorticoids administration due to severe pulmonary condition.

The VAS was significantly decreased postoperatively (6.5 [6–7] vs 0.5 [0–1]). For Caprini venous thromboembolism risk evaluation, 6 fracture patients were at “super high risk” level preoperatively, whereas all the 8 patients were at “super high risk” level after the procedure. However, no evidence of complications such as surgical wound infection, DVT, or dislocation was noticed till the last follow-up.

## 4 Discussion

The present study retrospectively reviewed the early clinical data of eight patients with COVID-19 or in the recovery stage of COVID-19 infection who received the joint arthroplasty procedure. The 2 patients with confirmed COVID-19 restored well from both the primary disease and the COVID-19 infection. Significant improved VAS was observed with no complications such as wound infection, dislocation, or DVT noted postoperatively. The 6 patients in the recovery stage also experienced an uneventful rehabilitation period. More importantly, there was no recurrence of the COVID-19 infection at the discharge. However, there was no evidence indicating COVID-19 infection in health care providers.

### 4.1 Patients with confirmed or highly suspected COVID-19 infection

For patients with confirmed or highly suspected COVID-19 infection, the joint arthroplasty could be performed in emergent cases under Level-II protection.^[2,4]^ The anesthesiologists should wear Level-III PPE with positive pressure headgear during anesthesia and intubation.^[6]^ In the present study, 2 patients with confirmed COVID-19 experienced a successful surgical and rehabilitation period. Patients were approved to mobilize 1 day after the surgery with weight-bearing as tolerated. The rapid pain relief and early reconstruction of the joint simplified the postoperative nursery process and enhanced the rehabilitation progress. It was reported that severe COVID-19-infected patients were at extremely high risk of lower extremity DVT^[17,18]^; however, there was no ultrasound detectable DVT noted in any of our patients during the follow-up. In summary, it could be postulated that COVID-19 patients with tolerable respiratory conditions for anesthesia could benefit from the joint arthroplasty procedure.

Concerns of PJI associated with COVID-19 infection were inevitable. Previous investigations documented that patients with pulmonary infection were at higher risk of PJI.^[19]^ However, no evidence of PJI was observed during our follow-up. Although the WBC and the CRP level were higher than the normal threshold preoperatively, they all came back to the normal range at the time of discharge, with the surgical wound healed uneventfully. We believed that COVID-19 infection did not directly lead to the early PJI after joint replacement, which might be related to the antibiotics used during the COVID-19 treatment. However, a longer-term follow-up was still in need to elucidate the issue.

During the post-outbreak period, preventing a second surge of COVID-19 in hospitals is paramount. The OR is a particularly important location for the prevention of further COVID-19 outbreak. To ensure the safety of the ORs, hospitals should provide sufficient prior storage and provision of protective supplies. Active cooperation by medical staff should be encouraged, and their physical and mental health status should be monitored.^[4]^ In the present study, although intraoperative osteotomy, reaming, and broaching process might generate aerosol during the procedure,^[13]^ the Level-II PPE could excellently protect the medical staff from SARS-CoV-2 infection, with no abnormal body temperature or any other related complaints such as cough, sore throat, or dyspnea observed in either the surgical team or anesthesiology team during the follow-up.

### 4.2 Patients in the recovery stage of previous COVID-19 infection

Some patients had successfully recovered from previous COVID-19 infection, with negative COVID-19 tests nor clinical and radiographic indications of COVID-19 infection; however, the anti-SARS-CoV-2 IgG could still be positive.^[20]^ As far as we concerned, the medical resources and PPE were relatively sufficient in the post outbreak period, and elective joint replacement surgery could be continued on the premise of appropriate protection. It has been reported that joint arthroplasty procedure belonged to Tier-2 surgery, of which the timing of the procedure should be postponed.^[2]^ However, many elderly patients were not “highly symptomatic” after the hip fracture,^[21]^ whereas some patients with osteoarthritis of the hip might have “symptomatic” complaints.^[22]^ Joint arthroplasty can relieve pain and improve joint function immediately, as well as their quality of life.^[8]^ In the present study, 6 previously COVID-19-infected patients in their recovery period successfully received joint arthroplasty procedures under Level-I protection. They could gradually proceed to the rehabilitation protocol under the supervision of orthopaedic surgeons and respiratory physicians after the operation. No recurrence of COVID-19 nucleic acid detection was observed and there was no evidence of PJI during the follow-up. Meanwhile, although only Level-I PPE was applied for the surgical and anesthesiology group, there was no indication of SARS-CoV-2 infection among the medical staff during the observation period.

The present study was not without limitations. It was a small and short retrospective observational study; thus, the results should be interpreted with cautious. For patients with confirmed, clinically diagnosed, or highly suspected COVID-19, we could not provide long-term rate of PJI and mortality, and a prolonged follow-up was still in need; however, a longer-term follow-up was under way to elucidate the issue.

The COVID-19 pandemic has a great and far-reaching impact on the global medical system.^[23]^ Orthopedic surgery, especially the joint arthroplasty procedure, has higher requirements for infection control, patient selection, and medical protection. We believe that for patients in recovery period who were only positive for anti-SARS-CoV-2 IgG, but were negative for nucleic acid test and typical clinical and pulmonary manifestations, an elective joint arthroplasty could be continued under comprehensive preoperative evaluation and appropriate medical protection. However, for patients who were tested positive for COVID-19 nucleic acid or whose clinical and pulmonary manifestations were still complicated, an elective joint arthroplasty could not be recommended. For such patients, the time of surgery might be considered to be postponed, and the operation should be carried out only after the patient's condition was clearly specified.

## Acknowledgments

The authors thank the participating patient and the contributions made by our colleagues.

## Author contributions

**Conceptualization:** Xin Jin, Xianzhe Liu.

**Data curation:** Xin Jin, Mengcun Chen.

**Formal analysis:** Xin Jin, Mengcun Chen, Jinlong Wang.

**Funding acquisition:** Xin Jin, Shuhua Yang.

**Investigation:** Mengcun Chen, Jinlong Wang.

**Methodology:** Xin Jin.

**Project administration:** Shuhua Yang, Xianzhe Liu.

**Supervision:** Shuhua Yang, Weihua Xu, Xianzhe Liu.

**Validation:** Mengcun Chen, Weihua Xu.

**Writing – original draft:** Xin Jin, Mengcun Chen, Xianzhe Liu.

**Writing – review & editing:** Xin Jin, Mengcun Chen, Jinlong Wang, Shuhua Yang, Weihua Xu, Xianzhe Liu.
